# Supplementary material for: Investigation of the Relationships between Coat Colour, Sex, and Morphological Characteristics in Donkeys Using Data Mining Algorithms
Source: Animals (Basel). 2023 Jul 20;13(14):2366. doi: 10.3390/ani13142366 (PMC10376350; doi:10.3390/ani13142366)
Supplement: Supplementary file 1 [file animals-13-02366-s001.zip › animals-2438262-supplementary.pdf]

Article

Investigation of the Relationships between Coat Colour, Sex, and Morphological Characteristics in Donkeys Using Data Mining Algorithms

Şenol Çelik [1,*] and Orhan Yılmaz [2]

[1] Biometry and Genetic Unit, Department of Animal Science, Faculty of Agriculture, Bingol University, Bingol 12000, Turkey
[2] Plant and Animal Production Department, Posof Vocational School, Ardahan University, Ardahan 75000, Turkey; orhanyilmaz@ardahan.edu.tr
* Correspondence: senolcelik@bingol.edu.tr

The Prediction Equation Results for the Bagging MARS Algorithm

```
> bagMARS$oob
     RMSE  Rsquared      MAE
[1,] 3.391163 0.7971010 2.712162
[2,] 4.582449 0.6737574 3.617525
[3,] 5.434235 0.5649297 3.374495
> cat(format(mu5))
  BL=(108.6688
  +   0.7055595 * h(WH-105)
  -   2.17283 * h(99-HR)
  +   0.1699517 * h(CC-107)
  -   1.899378 * h(41-HW)
  -   0.7495128 * h(HW-41)
  -   0.2444284 * h(50-TL)
  -   0.3697409 * Province Kars*h(105-WH)
  -   0.1295822 * h(105-WH)*h(EL-22)
  +   0.1273157 * h(HR-99)*h(29-CW)
  +   0.08126315 * h(104-HR)*h(41-HW)
  +   0.2053291 * h(HR-99)*h(39-TL)
  -   0.2801208 * h(HR-99)*h(52-HL)
  +   0.3064254 * h(41-HW)*h(HL-58)
  +   0.09016256 * h(41-HW)*h(58-HL)
  +   0.4655969 * h(41-HW)*h(CAC-13.5)
  +   0.04869532 * h(41-HW)*h(LL-45)
  +   0.2760453 * h(41-HW)*h(EL-22.5)
  +   0.1530143 * h(50-TL)*h(21-EL)
  -   0.1723079 * h(TL-50)*h(EL-22)
  +   109.2485
  -   0.9382122 * h(107-HR)
  +   2.483933 * h(HR-107)
  -   1.116736 * h(HW-41)
  +   1.615983 * h(38-TL)
  +   0.1763708 * h(TL-38)
  +   0.03688395 * h(101-WH)*h(107-HR)
  +   0.3488042 * h(WH-101)*h(107-HR)
  -   0.05538678 * h(107-HR)*h(107-CC)
  +   0.1822892 * h(107-HR)*h(CW-26)
  +   0.4428114 * h(107-HR)*h(26-CW)
  -   0.1194691 * h(HR-100)*h(41-HW)
  -   0.1066795 * h(107-HR)*h(51-TL)
  -   0.06071318 * h(109-HR)*h(TL-38)
  -   0.1032581 * h(HR-109)*h(TL-38)
  +   0.878669 * h(107-HR)*h(CAC-13.5)
```

+ 0.3322155 * h(107-HR)*h(13.5-CAC)

+ 0.06099415 * h(107-HR)*h(24-EL)

- 0.4135477 * h(26-CW)*h(41-HW)

- 0.09861111 * h(CW-26)*h(41-HW)

+ 0.2334746 * h(41-HW)*h(HL-59)

+ 0.03160098 * h(41-HW)*h(59-HL)

+ 105.1639

- 0.4696366 * h(104-WH)

+ 0.7602837 * h(WH-104)

- 3.231432 * h(CC-116)

+ 0.4090607 * h(118-CC)

+ 8.844629 * h(CC-118)

- 4.048582 * h(CC-119)

+ 0.2687515 * h(46-CD)

- 2.748363 * h(40-HW)

- 1.208723 * h(HW-40)

+ 1.640274 * h(TL-41)

- 1.713009 * h(TL-47)

- 1.948429 * h(47-LL)

- 0.5398618 * h(LL-47)

- 0.5592429 * Province Kars*h(104-WH)

+ 2.01843 * Province Mardin*h(40-HW)

- 1.217631 * ProvincVan*h(46-CD)

- 0.2142578 * h(2-Age)*h(118-CC)

- 0.04165114 * h(Age-2)*h(118-CC)

- 0.1174302 * h(6-Age)*h(CC-116)

+ 0.06116534 * h(Age-2)*h(40-HW)

- 1.017233 * h(2-Age)*h(41-TL)

+ 0.2189627 * h(Age-2)*h(41-TL)

+ 0.8046758 * h(104-WH)*h(CAC-13.5)

+ 0.2175535 * h(104-WH)*h(13.5-CAC)

- 0.03825487 * h(105-HR)*h(118-CC)

- 0.07320834 * h(HR-105)*h(118-CC)

+ 0.1669249 * h(112-CC)*h(CD-46)

- 0.06852234 * h(CC-118)*h(TL-43)

- 0.1997813 * h(CC-118)*h(43-TL)

+ 0.4741123 * h(118-CC)*h(EL-24)

- 0.05721887 * h(118-CC)*h(24-EL)

- 0.1610904 * h(118-CC)*h(EL-22)

+ 0.0742018 * h(40-HW)*h(59-TL)

+ 0.06577163 * h(40-HW)*h(57-HL)

+ 0.198802 * h(40-HW)*h(LL-48)

+ 0.2341542 * h(40-HW)*h(48-LL)

- 1.166718 * h(39-TL)*h(LL-47)

```
) / 3
> mu5$fit
$Resample1
Selected 20 of 36 terms, and 11 of 29 predictors
Termination condition: Reached nk 36
Importance: HR, WH, HW, CAC, HL, TL, EL, ProvinceKars, LL, CW, CC, ColorBlack-unused,
ColorBrown-unused, ColorGray-unused, ...
Number of terms at each degree of interaction: 1 6 13
GCV 11.58879    RSS 3248.805    GRSq 0.777539    RSq 0.8309909
$Resample2
Selected 22 of 27 terms, and 9 of 29 predictors
Termination condition: RSq changed by less than 0.001 at 27 terms
Importance: HR, HW, CW, HL, CAC, TL, WH, CC, EL, ColorBlack-unused, ColorBrown-unused,
ColorGray-unused, ColorWhite-unused, ...
Number of terms at each degree of interaction: 1 5 16
GCV 11.74647    RSS 3191.692    GRSq 0.7745919    RSq 0.8340208
$Resample3
Selected 38 of 51 terms, and 14 of 29 predictors
Termination condition: Reached nk 59
Importance: HW, WH, CAC, ProvincKars, TL, ProvincMardin, Age, CC, EL, ProvincVan,
CD, LL, HR, HL, ColorBlack-unused, ColorBrown-unused, ...
Number of terms at each degree of interaction: 1 13 24
GCV 6.660898    RSS 1382.563    GRSq 0.8669723    RSq 0.9251719
```
